# Data and Digital Solutions to Support Surveillance Strategies in the Context of the COVID-19 Pandemic

**DOI:** 10.3389/fdgth.2021.707902

**Published:** 2021-08-06

**Authors:** Patty Kostkova, Francesc Saigí-Rubió, Hans Eguia, Damian Borbolla, Marieke Verschuuren, Clayton Hamilton, Natasha Azzopardi-Muscat, David Novillo-Ortiz

**Affiliations:** ^1^UCL Centre for Digital Public Health in Emergencies (dPHE), Institute for Risk and Disaster Reduction, University College London, London, United Kingdom; ^2^Faculty of Health Sciences, Universitat Oberta de Catalunya, Barcelona, Spain; ^3^Interdisciplinary Research Group on ICTs, Barcelona, Spain; ^4^SEMERGEN New Technologies Working Group, Madrid, Spain; ^5^Department of Biomedical Informatics, University of Utah, Salt Lake City, UT, United States; ^6^Division of Country Health Policies and Systems, Regional Office for Europe, World Health Organization, Copenhagen, Denmark

**Keywords:** digital surveillance, digital epidemiology, data sources, outbreak, COVID-19

## Abstract

**Background:** In order to prevent spread and improve control of infectious diseases, public health experts need to closely monitor human and animal populations. Infectious disease surveillance is an established, routine data collection process essential for early warning, rapid response, and disease control. The quantity of data potentially useful for early warning and surveillance has increased exponentially due to social media and other big data streams. Digital epidemiology is a novel discipline that includes harvesting, analysing, and interpreting data that were not initially collected for healthcare needs to enhance traditional surveillance. During the current COVID-19 pandemic, the importance of digital epidemiology complementing traditional public health approaches has been highlighted.

**Objective:** The aim of this paper is to provide a comprehensive overview for the application of data and digital solutions to support surveillance strategies and draw implications for surveillance in the context of the COVID-19 pandemic and beyond.

**Methods:** A search was conducted in PubMed databases. Articles published between January 2005 and May 2020 on the use of digital solutions to support surveillance strategies in pandemic settings and health emergencies were evaluated.

**Results:** In this paper, we provide a comprehensive overview of digital epidemiology, available data sources, and components of 21st-century digital surveillance, early warning and response, outbreak management and control, and digital interventions.

**Conclusions:** Our main purpose was to highlight the plausible use of new surveillance strategies, with implications for the COVID-19 pandemic strategies and then to identify opportunities and challenges for the successful development and implementation of digital solutions during non-emergency times of routine surveillance, with readiness for early-warning and response for future pandemics. The enhancement of traditional surveillance systems with novel digital surveillance methods opens a direction for the most effective framework for preparedness and response to future pandemics.

## Introduction

In London's Soho district in 1854, the father of public health John Snow removed the handle of the local community water pump to stop the spread of the famous cholera outbreak (1). Snow proved cholera is a water-borne disease rather than an airborne “bad air” infection by manually mapping data of citizens infected and deaths onto a map (2). This began what we today call epidemiology and surveillance.

Public health surveillance is a regular routine process of collecting data on diseases, cases and public health interventions (such as vaccination) to inform public health authorities about the situation in order to respond with appropriate public health measures. Public health surveillance also includes early warning systems alerting about upcoming outbreaks and emergencies. In order to enable rapid response, inform public health policy and strategies (3). The recent increase of big data and digital and mobile technology has enabled the rapid growth of “digital epidemiology.”

Digital epidemiology provides numerous opportunities and challenges and now is an indispensable part of infectious disease surveillance systems. Epidemic intelligence is understood as the systematic collection and analysis of traditional (indicator-based epidemiological) and new data sources (event-based surveillance), which are used to identify new infection threats to provide early warning and rapid assessment of risk (4). Once a threat or an outbreak is detected and an event verified and risk assessed, a rapid response must be implemented to control the outbreak, including diagnosis, testing, contact tracing, and risk communication with the public.

These novel data sources arise from new digital solutions such as tracking devices, mobile applications (apps), and social media interventions; they can also contribute to infectious disease outbreak management. Digital epidemiology uses these devices and the data gathered to find new solutions to minimise disease spread as well as determine the population's behaviour and insights, e.g., behavioural reactions to public health interventions, contact tracing, and others (5). However, considerable computational and technical challenges arise from the rapid increase in relevant data from digital data sources: “Extracting meaningful information from this data deluge is challenging, but holds the unparalleled potential for epidemiology” (6). The general term for analysing and disseminating real-time health information from news and social media is also referred to as “infodemiology” (7, 8).

With the amount of data that can be collected through digital epidemiology, making sense of the data and determining whether it will adequately support epidemiological surveillance can pose difficulty. Applications and digital tools are being developed to process large volumes of unstructured data (big data) to help uncover useful information for problem solving. The term “big data” refers to the use and analysis of verified information that has been collected. This includes complex data that is rapidly collected in massive amounts, as long as the data is real and verifiable (9, 10). Digital sources of big data in healthcare include electronic medical records, genomics, imaging data, data from social networks, and sensor data (11).

Big data can be extracted from diverse real-time or static information sources that are often underutilised or not accessible, which could potentially increase the acquisition of new knowledge contributing to a better understanding of disease epidemiology. Algorithmic analysis to “train” data for classification or predictions for decision-making is a rapidly growing computer science domain called machine learning.

Digital epidemiological surveillance involves sources that are not typically used in traditional epidemiology, generating larger amounts of information that should be incorporated into public health systems as part of the response to traditional diseases, new emerging pathogens such as the COVID-19 virus we are currently fighting.

The main aim of this paper is to provide a comprehensive overview for the literature digital solutions and big data to support surveillance strategies in the context of the COVID-19 pandemic and beyond.

## Methods

This research is a review of original research on digital surveillance from January 2015 until May 2020 with implications for opportunities and strategies for new outbreaks such as the COVID-19 pandemics and future emergencies.

A review of literature going back 15 years was conducted as 2005 was chosen as the year of the dawn of wide spread mobile technology and big data. We focused on syntesis of aproaches in order to draw implications for surveillance opportunities for COVID-19 and beyond (we are aware this study is not presenting a review of COVID-19 strategies as it is too soon to conduct such an excercise).

The search was conducted in the electronic database MEDLINE (accessed by PubMed) for articles published between January 2005 and May 2020 using combinations of the following free terms and Boolean operators (AND and OR):

“COVID-19”[Title/Abstract] OR “COVID-19 diagnostic testing”[Supplementary Concept] OR “surveillance”[Title/Abstract] OR “Pandemics”[MeSH Terms] OR “epidemic control”[Title/Abstract] OR “self-diagnosis”[Title/Abstract] OR “self-evaluation”[Title/Abstract] OR “contact tracing”[Title/Abstract] AND (“digital health”[Title/Abstract] OR “information system^*^”[Title/Abstract] OR “apps”[Title/Abstract] OR “eHealth”[Title/Abstract] OR “e-Health”[Title/Abstract] OR “electronic health record^*^”*[Title/Abstract] OR “big data”[Title/Abstract] OR “machine learning”[Title/Abstract] OR “data science”[Title/Abstract] OR “artificial intelligence”[Title/Abstract] OR “mHealth”[Title/Abstract] OR “m-Health”[Title/Abstract] OR “social media”[Title/Abstract] OR “IoT”[Title/Abstract] OR “smartphone”[Title/Abstract] OR “Internet of things”[Title/Abstract])*.

The search was limited to English-, Portuguese-, and Spanish-language publications and was complemented using the snowballing technique to identify relevant articles in the reference lists of articles returned by our search (12). Additional search for grey literature was conducted regarding digital surveillance. Expanded grey literature searching included internet search engine, targeted websites and social media. The search is subject to a selection bias as publications were limited to the three major languages, however, as the majority of scientific literature is published in the three major languages this bios is minimal.

Diverse studies covering the use of digital data sources for surveillance during a health emergency were included. Initial screening was based on titles and abstracts, and articles were independently evaluated. Abstracts lacking sufficient information to identify their inclusion or exclusion, were retrieved for full-text evaluation. Subsequently, two investigators independently evaluated the full-text articles and determined eligibility for inclusion or exclusion. Authorship, journal, or years were not blinded.

### Study Selection

The initial research included complete publications and abstracts that were reviewed to determine whether they met the inclusion or exclusion criteria. Abstracts lacking information were retrieved for full-text evaluation. The inclusion criteria were (1) original research articles, (2) studies conducted during outbreaks or pandemic situations that measured the use of digital tools for contact tracing, (3) studies on the application of data and digital solutions to support surveillance strategies, and (4) studies covering the use of digital data sources for surveillance during a health emergency. The exclusion criteria were studies that described the use of technology outside an epidemic, big data studies that were not focused on epidemiological problems, and other surveillance interventions that were not related to the use of digital health solutions.

**Figure 1:**
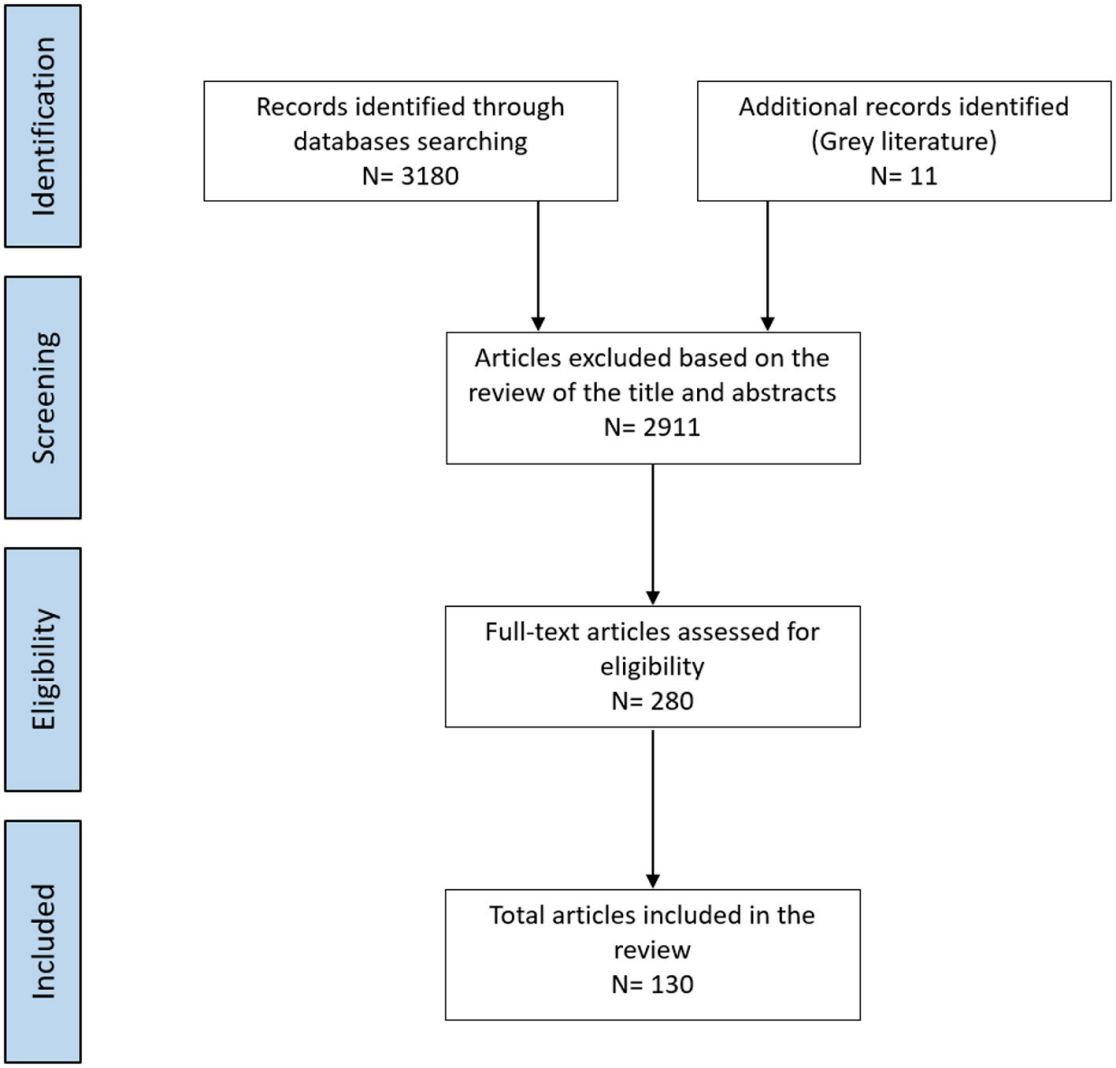
Flow diagram of studies through the review.

After the first review of the titles and abstracts, 280 studies were selected. For the grey literature, 11 electronic notes were reviewed. After reading the full texts of these studies, 130 were deemed to have met the search selection criteria, one of them was grey literature. Two authors (DB and FSR) screened all articles individually. Discrepancies were resolved through discussion with a third author (DN) when necessary. All the data were analysed qualitatively and quantitatively. The search and selection processes are summarised in Figure 1.

Based on the surveillance and early-warning & response processes established at WHO, ECDC and member states, four core components of digital epidemiology were identified in previous research and expanded^1^:

- digital surveillance, supporting public health routine surveillance;
- early warning and epidemic intelligence, striving to improve early warning tools that alert public health professionals to upcoming threats. After a threat has been verified, big data analysis might support public health experts in:
- rapid response, outbreak control, and increasing the impact of public health measures through digital interventions; and
- risk communication and public communication being advanced through mobile apps and social media, while the improvement in epidemic modelling that leverages real-time heterogeneous data improves public health policy through better assessment of how control measures impact healthcare and society.

While our systematic review illustrates how digital technology has been contributing to these four subdisciplines for over a decade. In Table 1, we map them to the objectives of COVID-19 public health surveillance defined by World Health Organization (WHO) (13)—requiring a predominantly public health and healthcare sector response—complemented by opportunities provided by digital epidemiology leveraging novel big data streams.

**Table 1:** Mapping of four digital epidemiology disciplines to WHO objectives of COVID-19 surveillance.

**The objectives of COVID-19 surveillance**	**Sub-disciplines of digital epidemiology**
“Enable rapid detection, isolation, testing, and management of suspected cases”	Early warning and Epidemic intelligence digital surveillance
“Monitor trends in COVID-19 deaths”	Digital surveillance
“Identify, follow up and quarantine of contacts”	Digital surveillance
“Detect and contain clusters and outbreaks, especially among vulnerable populations”	Rapid response, outbreak control digital interventions
“Guide the implementation and adjustment of targeted control measures, while enabling safe resumption of economic and social activities”	Rapid response, outbreak control digital interventions
“Evaluate the impact of the pandemic on health-care systems and society”	Public communication and the impact on healthcare and society
“Monitor longer-term epidemiologic trends and evolution of SARS-CoV-2 virus”	Digital surveillance
“Contribute to the understanding of the co-circulation of SARS-CoV-2 virus, influenza and other respiratory viruses, and other pathogens”	Digital surveillance

While reviewing the articles using an iterative process (with regular meetings), the digital data and innovation systems were described in line with the four digital epidemiology categories.

## Results

Our results provide an overview of the literature identified throughout our search process and highlight the existing opportunities and applications for the found disciplines in digital epidemiology.

### Digital Surveillance

Surveillance is a core component of public health and preventive medicine and can be categorised as “active” (where health authorities make direct contact with the population or care providers to measure the actual conditions) or “passive” (when the health authorities get pre-designed reports about specific conditions, typically with the care providers reporting) (14).

With the use of new technology—for example social networks (15)—the traditional surveillance process can be not only enhanced but also reach a wider population (16). Passive surveillance, drawing from a wide range of novel data sources, is less accurate and subject to elevated noise in the data, either by proactively searching related information and or by gathering it passively, making it less resource intensive (17).

Before an infectious disease is confirmed by a laboratory, infected persons may exhibit symptoms, signs, behavioural patterns, or laboratory findings that can be tracked through a variety of data sources (18). This relates to what is called “syndromic surveillance,” which may be boosted by the use of digital technologies.

Integrated or enhanced surveillance combines both systems (19): traditional and digital. The strength of syndromic surveillance systems is in early warning systems for emerging disease threats, such as dengue (20), while the strength of indicator-based (active) surveillance is the production of regular, robust, reliable surveillance reports (21). Syndromic surveillance threats could also capture undiagnosed infections (22, 23). A good example is metagenomics, as infected (and infectious) people who are asymptomatic may unwittingly spread the infection to others (24).

The use of online, mobile, and social media data streams for routine surveillance enhances traditional methods (3, 25). Also called “participatory surveillance,” (26)—disease and symptoms reported directly by citizens themselves rather than by health authorities—the new generation of health trackers and sensors brings new opportunities (5). In this type of surveillance, the structured data collection across all the participating countries enable study and research of subgroups, e.g., the health status of the population outside the health care system (27, 28) or vaccine effectiveness in vaccinated groups and attitudes towards vaccination (such as influenza vaccination) (29, 30). The desirable benefits for participants, improving the long-term engagement in standard reporting, include real-time information on disease rates at local or neighbourhood levels (31).

Participatory systems have also been successfully implemented for mapping tropical diseases such as Zika and dengue and during mass gatherings like the 2016 Olympics in Rio de Janeiro, Brazil (32). Such a “hybrid” system (3, 33) (for example, using online surveys, communicating events, or using websites and applications) was successfully explored by combining Zika-related Google searches, Twitter microblogs, and the HealthMap digital surveillance system (34, 35) and cross-validating them with traditional disease surveillance data (36). Combining diverse data sources, search queries, social media data, digital data from internet-based sources (25), and website visits have proven effective for digital surveillance systems (37). With a new generation of trackers and sensors, this kind of individual surveillance will soon increase in scope, intensity, and significance (5), especially for emerging diseases and epidemic outbreaks, helping to monitor populations and potential security threats (5). Online surveillance-mapping tools have the potential to improve the early detection of infectious diseases compared to traditional epidemiological tools (38).

Online surveillance-mapping tools used in tandem with traditional epidemiological tools can improve the early detection of infectious diseases as was accomplished with Ebola (38) and Zika with critical results. Mobile phone data was used in Kenya to help identify the dynamics of human carriers that drive malaria parasite importation between regions (39); Google Trends was used for epidemiologic searching for Mayaro virus (40). All of these technologies are being employed for the COVID-19 epidemic (41). Mobile phone data and apps where users can report cases are also valuable tools for surveillance purposes (42). Due to the universal ownership of mobile devices with Bluetooth, these types of applications can be used all over the world, including low-connectivity and low-resource environments like rural Sierra Leone (43).

Many other instances of the digital surveillance of emerging infectious diseases have successfully used digital media for public health management (44, 45), including dengue (46), chikungunya (47), Ebola (48), monkeypox (49), and influenza (3, 50). The most common disease surveillance that utilises social media analytics for early detection and surveillance is influenza (51). For example, digital surveillance in influenza was used in Canada to demonstrate the correlation between influenza incidence and Google Ads click rates (52). It was also used to find the correlation between influenza incidence and Yahoo search trends (53). It is not only used at a national level but also in regional and local areas (54, 55).

Many examples show digital surveillance and epidemic intelligence noticeably using social networks, indicating the essential role social media plays, even according to the World Health Organization (WHO), for which more than 60% of initial disease epidemic reports derive from unofficial sources (56). Twitter, for instance, is used for surveillance because it can be used in outbreaks or emergencies, in monitoring diseases, in prediction, in gauging public reactions, in lifestyle analysis, in geolocation, and other general applications (57). Twitter was also used for Middle East respiratory syndrome surveillance in Korea (58), to analyse the H1N1 pandemic in 2009 (59), and to broadcast information about Ebola (60). In the literature, infectious disease surveillance, predicting disease spread, dissemination of public health information, and assessment of the public's views on public health outbreaks are some of the roles suggested for Twitter (34, 45, 51, 57–60).

Studies on digital information communication on social media sites are on the rise, as they have played an important role in real-time analysis and have been used for faster trend prediction (61); however, these studies are often small pilots that need more methodological rigour and scalability (62). More studies should be conducted using appropriate technologies such as web-based systems to help support data quality improvements and future reporting (63).

### Early Warning and Epidemic Intelligence

Digital surveillance expands traditional epidemiology by adding information that previously did not exist. Important new sources of data include social networks, geographical location measured by GPS, wish lists, and consumption of mobile data, etc. These new types of information are not medically related (the classic epidemiological domain) but allow the expansion of early warning and prevention systems to prevent avoidable exposure to public health threats (64). Systematic review of digital data source their implication for health could be found in Li et al. (65)—some examples of novel data sources for the news of surveillance and epidemic intelligence are:

**Online Media:** Screening online news for mentions of specific diseases or conditions could be helpful to identify, for example, local food poisoning outbreaks. Special systems such as GPHIN (66), WHO EOIS (67), and HealthMap (68) screen all global media in multiple languages and were developed specifically to monitor epidemic events.
**Online Searches:** Search terms provide an invaluable geo-located monitoring tool for public information that could reveal public sentiment, shopping panics, or disease outbreaks, as demonstrated by Google Flu Trends in 2008 (40). While the opportunities are vast, commercial ownership of the search engines by Google and other tech giants prevents researchers and public health experts from exploring this resource for public health purposes. Online searches were also analysed on public medical websites such as the National Electronic Library of Infection and National Resource for Infection Control (69) to identify spikes in information needs resulting from the publication of major government guidelines (70).
**Sensors, Digital Traces, and Internet of Things (IoT) Devices:** Monitoring of population movements through citizens' digital traces via GPS-enabled phones, sensor networks, and credit/store cards seamlessly collects information about our moves, physical locations, purchases, online preferences, and payments and could provide early warnings of upcoming outbreaks. However, like Google, these datasets are mostly owned by commercial companies (supermarkets, pharmacies, etc.) and are not available for research or public health benefits.
Internet of things (IoT)-enabled devices and sensors allow real-time data streams from readings and measurements of environmental or smart home devices and could create opportunities for digital epidemiology such as mapping the spread of infection (71).
**Mobile data and Mobility GPS data**: Medical apps and games and health condition tracking devices support citizens in activities such as managing long-term conditions, increasing their physical activities, or losing weight (72) but create ethical challenges (64). GPS location and mobility data also play a part in locations and directions (navigation and mapping apps), or contact tracing (e.g., COVID-19 contacts) using Bluetooth, GPS, cellular location tracking and QR codes (73). For example, geo-coded electronic health records (EHR) were successfully mapped to better visualise the spread of methicillin-resistant Staphylococcus aureus (CA-MRSA), identifying risks for CA-MRSA in children that would not have been uncovered using traditional EHR analyses (74). For accuracy and privacy, Bluetooth technology with a decentralised server architecture is recommended (75). Bluetooth, however, is not precise enough to avoid false positive contacts (10).
**Social Media Streams**: Unlike digital traces, collected seamlessly, the increase of Web 2.0, user-generated content actively shared via social networking tools, has seen an unprecedented explosion. The privacy settings of Facebook, Instagram, and other social networks allow users to restrict their profile content and activity. Consequently, the most important social media channel for research has undoubtedly become Twitter due to a relatively open data policy that allows researchers and IT developers access to tweets through an open, free API, returning a 1% random sample of raw tweets free of charge.

The use of social network data can also provide early warning because analysis can detect a peak in an outbreak up to 2 weeks before the official public health authorities (as occurred in 2009 for swine flu) (76). In 2019, earlier than the official reports, Twitter reported almost one-third of the total notifications related to avian influenza outbreak (77). This corroborates that social networks can serve as a method to obtain valuable information on a disease's behaviour or spread, even a week in advance of what the general practitioners in a particular locality could report (78). The evaluation of commonly used drugs for seasonal influenza on Twitter also provides surveillance ahead of the flu season (79).

Social media data allow disease tracking (80, 81) and can help make predictions that could prevent danger to the population (3). Social media analytics in correlation with traditional laboratory data can predict an outbreak; examples of this include the cases of influenza and cholera (82) or Ebola and Marburg filoviruses (83). Some researchers also conducted a content analysis to identify key trends during the 2009 H1N1outbreak that could also correlate with outbreak incidence data (84). Even concerning the coronavirus, Kogan et al. found that Twitter could be used as a kind of barometer, showing potential growth 2–3 weeks before the growth of coronavirus cases (by region) (85) and in other cases even 8–12 days before the outbreak (86). This is not new. An analysis of more than 500 million tweets worldwide found a significant association between the geographic locations of HIV-related tweets and HIV prevalence (87).

Twitter data in 2010 accurately tracked the spread of cholera, but researchers advised that this type of information is not always reliable and must not replace traditional epidemiological methods, as information and guidance is missed (88). Moreover, social network analyses need to be challenged and scrutinised by the “ground truth.” In 2016, Mowery et al. conducted a study describing how epidemiological surveillance of influenza using Twitter incorrectly predicted the 2011–2012 flu season 3 months early (89).

Improvements in services and cost reductions in the health sector coupled with the need for early warnings for the onset of adverse health conditions are the main drivers of these developments and new sources of data (90). These novel data sources collect a massive amount of new information, and it can be difficult for big data tools to review the data obtained and present it as new useful knowledge for the prevention of a pandemic outbreak. However, the use of big data is proving crucial for the COVID-19 pandemic (91).

### Rapid Response, Outbreak Control, and Digital Interventions

Once a threat or an outbreak is detected, an event verified, and risk assessed, a rapid response must be implemented to control the outbreak, which ranges from diagnosis, testing, and contact tracing to risk communication with citizens.

This iterative process includes discovering patterns and generating new information (92) that can be used to control the outbreak. Digital information and new technologies are providing a quick response and coordinated control and management of possible outbreaks. Through monitoring cases using mobile technology, contact tracing infected citizens, following up with patients, and providing medical advice, digital and mobile technology can successfully complement the efforts of medical and public health experts (93).

With social networks, big data enables the early epidemiological storey of an outbreak to be reconstructed (94). Data streams and non-classical datasets in the early stages of the outbreak can inform the design and implementation of effective public health measures (95).

Big data could effectively support the rapid response to and better control of an outbreak but could do so more quickly and generate more accurate predictions. The algorithms that accomplish this are known as artificial intelligence (AI). For example, Toronto's surveillance system was first to detect the COVID-19 epidemic outbreak in the first reported epicentre of Wuhan (96). Also, scientists from the John Hopkins University visualised the spread of the coronavirus in real-time (95). AI is changing the landscape in public health and clinical management with very promising results (97). For example, a project seeking information related to the COVID-19 called Evidence Navigator provides computer-generated evidence maps of scientific publications on the pandemic, which is updated daily in PubMed (98).

Machine learning (ML) is a dramatically growing computer science AI discipline investigating algorithms to find new results or predictions without looking for specific solutions. ML was used to analyse several projects using the internet to enhance epidemiological surveillance and disease prediction [e.g., malaria (99), dengue (100), and influenza (101)]. The research demonstrated a positive predictive value of the incidence of infectious diseases (101) or little predictive value (102). ML was successfully used to create an evidence-based guideline from the information gathered from the Ebola virus epidemic and turn it into an application called the Ebola Care Guidelines app (103) to inform the general population and healthcare providers of updated, evidence-based guidelines in real time during a global pandemic (41).

In Africa, the rapid recognition of localised areas of higher transmission of Ebola and the resulting quantitative assessment could support the optimal deployment of public health resources (104). In Latin America and the Caribbean, the rapid integration of Zika virus prevention recommendations into sexual and reproductive health services made it possible to reduce the incidence of the Zika virus (105). In 2012, digital pens were used by the New Hampshire Department of Health and Human Services to rapidly acquire epidemiologic data during a gastrointestinal illness outbreak (106), providing rapid assessment, response, and control measures before the problem multiplied.

The novel digital information gathered about the coronavirus is being used in Taiwan in conjunction with their immigration database to classify travellers according to different risk types and to issue alerts in real time to prevent infections (107). This is often called a “digital fence.” Russia, China, and Poland have used facial recognition software to monitor population compliance with government policies (42). Applications exist for self-testing, quarantine monitoring, and contact tracing that are being already used in many parts of the world such as India to support the rapid response to COVID-19 (108).

Online news surveillance and mapping tools have successfully provided early warnings, but their potential to improve response and risk assessment as the outbreak progresses over time has led to the adoption by WHO operations of the most robust systems: e.g., GPHIN, Medisys, SORMAS (Surveillance and Outbreak Response Management and Analysis System) (109) and HealthMap (68). News surveillance also means that real-time feedback and effective responses should function as an intervention. Locally appropriate technologies, such as web-based systems and mobile phones, can help support data quality improvements and reporting timeliness (63).

In response to the COVID-19 outbreak, the rapid development and deployment of point-of-care (POC) diagnostics for screening have shown to help slow the spread of the disease (110), demonstrating that telemedicine can be used as a means of surveillance. For example, Botswana is using telemedicine to control patients remotely (111), and obstetric departments in the US are monitoring coronavirus patients (112). Rapid deployment of an in-patient telemedicine response is feasible across many settings in response to the COVID-19 pandemic (113).

### Public Communication and Evaluation of the Impact on Healthcare and Society

An essential piece at the centre of outbreak response success is public communication: a clear and concise message communicating the risk, measures, and policies taken by the government. Digital technologies complement traditional mass media and play an increasingly important role for sharing reliable, evidence-based information with the public and gaining citizens' buy-in (114).

While traditional media channels—newspapers and television—are still actively used for mass communication, the role of social media has grown dramatically. In particular, Twitter has demonstrated great potential to be used not only for tracking epidemics but also to inform citizens about the risks of pandemics in real time (57). Social media can be also used to study the public's risk perceptions (115). Mobile apps and wearable devices have further been used to monitor patient behaviour to provide personalised service and advice (116, 117). Twitter was analysed for risk communication potential during the epidemics of the Middle East respiratory syndrome (58), SARS (45), Ebola virus (60), Zika virus (34), H1N1 (“swine flu”) (59), and H7N9 (“avian flu”) (62). A study in Vietnam includes social media and science journalist for COVID-19 public policy^2^. The studies involving Twitter and other social networks summarise how they could be useful tools for disseminating information to the population about how to avoid the spread of the outbreak (118).

Specifically, role-play social media, a channel for gauging public attitudes, could effectively be used to disseminate risk communication in real time (78) but could be a double-edged sword in that also it is prone to misuse, misinformation, and the spread of fake news. How online channels inform healthcare professionals and the public and their genuine information needs to be part of any comprehensive government communication plan (70).

## Discussion

The results have shown many opportunities ranging from the use of social networks to the use of AI and big data for digital surveillance and reference early warning and epidemic intelligence, rapid response, outbreak control, risk communication, and public communication. The results present solutions that have been implemented in many countries with differing results because of their diverse socio-cultural realities. In August 2020, WHO updated the objectives for COVID-19 surveillance: (i) enable rapid detection, isolation, testing, and management of cases; (ii) monitor trends in COVID-19 deaths; (iii) identify, follow-up, and quarantine of contacts; (iv) detect and contain clusters and outbreaks, especially among vulnerable populations; (v) guide the implementation and adjustment of targeted control measures while enabling safe resumption of economic and social activities; (vi) evaluate the impact of the pandemic on healthcare systems and society; (vii) monitor longer term epidemiologic trends and evolution of SARS-CoV-2 virus; and (viii) contribute to the understanding of the co-circulation of SARS-CoV-2 virus, influenza and other respiratory viruses, and other pathogens (119).

With digital epidemiology's subdisciplines already assessed and ready for use, the population must be made aware of the measures to be followed to control the outbreak using (ix) public communication and evaluation of the impact on healthcare and society as culture might shape how people think about privacy and surveillance Risk communication and public communication are now taken to a new level through mobile apps and social media, while the advances in epidemic modelling that leverage real-time heterogeneous data improve public health policy through better assessment of the impact of control measures on healthcare and society.

Mobile applications and mHealth approaches could be used for public health surveillance due to their multiple benefits as an efficient, almost universal presence (120), a contributor to high digital literacy, and a source that creates wide availability of data. With the number of mobile phones around 14 billion units worldwide and expecting an increase to almost 17 billion by 2023, the options available with this type of device must be considered. The use of mobile phones and Bluetooth to assess the follow-up of potential patients has always been a strong alternative (121). Apps with tracing functionalities using Bluetooth technology can support health authorities in the contact tracing process, identifying the possible contacts (known or unknown) of a confirmed/positive case, creating a network that will function as epidemic control if it is used by enough people (122). Some examples exist of mobile apps and mHealth being used already. For example, the Chinese government required citizens in 200 cities to use the Alipay app, assigning a risk code (green, yellow, red) to each person indicating to what extent they were allowed to move around the community. An algorithm incorporated information about the time they spent in risky locations and the frequency of contact with other people (42).

Although these technologies only use monitoring data temporarily, many people are reluctant to use them because they think that their data will be sold to private companies or they will continue to be monitored once the pandemic is over (123). Therefore, notification is necessary that the data obtained will be used only for monitoring purposes, that it will not be transferred to any public or private company, and that it will only be used in periods of a pandemic or used as anonymised data (124).

Mobile devices (including tablets, wearables, etc.) also can connect to social networks, which allows their users to be informed of the latest news about the pandemic. At the same time, they can be used to provide measures to avoid contagion. Social media's power of influence is vast, becoming an important tool public health during a pandemic, which is precisely a difference between digital and traditional epidemiology. Social networks can provide a different perspective than the traditional approach that relies on health reports by providing important correlations with abnormal disease trends that could indicate a potential outbreak.

The use of social media also offers significant opportunities to encourage citizen engagement in crisis management (125). However, not all the most-read information on social networks is true. Many celebrity “influencers” (people with many followers, such as actors, athletes, etc.) publish controversial posts such as false cures or prophylactics for the coronavirus or indicate that it is simply an invention (126). This is due to voluntary submission and a lack of gatekeeping (127). The advances in AI could help in this facet (128). This kind of digital technology, correctly applied, could benefit the healthcare landscape in public health and clinical management with promising results (97). It has already been used for some time to handle data related to the coronavirus (96).

Big data use for surveillance seems to be a beneficial tool for public health but it must follow a rigorous statistical analysis. Qin et al. used social media search indexes (SMSI) to predict the new number of COVID-19 cases that could be detected 6–9 days in advance (129), but the prediction is generated after an algorithm has evaluated the information. With all this generation of digital information, there is a risk of losing what is impactful to the epidemiological study. To accomplish an “understanding of all data” created by social networks, the use of big data has also been implemented.

While useful in digital epidemiology, big data can also serve as a standard to control possible adverse effects and can support traditional epidemiology by discovering additional facts from social media behaviour that may complement other data from the population. Furthermore, it can help overcome some challenges, such as geographic heterogeneity, insufficient representation in developing countries, and spatial/temporal uncertainty in the information obtained. On the other hand, a disadvantage of using internet search data or data from social networks for surveillance purposes is its—eventual—lack of representativeness, as well as possible fake results. In order to create big data, information must be collected and processed before it can be used; therefore, the WHO has called for requiring the detection and management of suspicious cases at entry points worldwide to generate this information to avoid the spread of COVID-19 (130). Information from symptoms checkers and laboratory data could help to estimate the predictive value of the respiratory symptoms on the community as well as present facts on the level of virus circulation (93).

The use of this technology would allow epidemiologists to evaluate millions of digital trails of people who constantly use their digital equipment such as mobile phones for social networking. However, an ideal prediction using machine learning and AI is not yet realised; the tracking and prediction of how COVID-19 will spread are not yet completely reliable. This could be due to two reasons: (1) the availability of COVID-19-related clinical data is a key barrier; and (2) AI requires data on COVID-19 to train itself (131). Therefore, the WHO recommends being cautious with the implementation of digital solutions until the utility of public health policies are better understood. Planned solutions should cover and allow (i) quality monitoring, transparency, and accountability, (ii) resource allocation optimisation, (iii) citizen participation and inclusion, and (iv) resilience and adaptation to exogenous events.

Several promising initiatives have been launched to gather and share both existing and new data using new AI models, including WHO's Global Research on Coronavirus Disease Database, along with the GISAID Initiative (Global Initiative on Sharing All Influenza Data); the COVID-19 Open Research Dataset Challenge of Kaggle data science platform; the around 20,000 related articles in ScienceDirect in its Novel Coronavirus Information Centre early-stage and peer-reviewed research on COVID-19; etc. Finally, the initiative formed by Microsoft, Facebook, Semantic Scholar, the Allen Institute for AI, and five other collaborators to make the COVID-19 Open Research Dataset (CORD-19) openly available, which contains about 44,000 scholarly articles for data mining, is worth mentioning.

Finally, there are a number of related subjects that are beyond the scope of this paper. Firstly, cost issues and culture aspects of surveillance were not part of our initial search strategies, however, it is important to note the cost to setting up and conducting digital surveillance, especially in low and middle income countries^4^,^5^. Culture attitudes (positive or negative) towards digital surveillance are also essential to consider for a successful deployment of a digital solution^6^,^7^ as demonstrated in fight against COVID-19 in Vietnam^8^. Final limitation of this study includes our search strategy focusing on PubMed and therefore not covering studies published in computer science outlets such as conferences published in ACM and IEEE libraries, such as pioneering Twitter research for epidemic intelligence and eastly warning (see text footnote 1)—this is a subject for a future research.

## Conclusions

We have highlighted the opportunities and challenges for digital epidemiology as a growing discipline that have contributed to surveillance of the COVID-19 pandemic and highlighted how digital epidemiology will become indispensable for fighting future public health and natural disasters and pandemics.

Prevention and control of the COVID-19 pandemic requires public health and epidemiology measures. The use of digital technology enhances traditional epidemiological means to contain outbreak and supports prevention, early warning, rapid response, and digital interventions such as remote care for patients or providing reliable information to the public. In addition, it is crucial that the technology is inclusive and user friendly (for example, social networks and specially designed apps). However, additional support strategies are required for vulnerable groups who are not active technology users.

The key opportunities and challenges for effective digital epidemiology systems for the 21st century lie in front of us. To improve capacity and preparedness for later epidemics, the repurposed and emerging systems for COVID-19 need to be fully developed and evaluated after the crisis with the goal of creating fully integrated, interoperable digital epidemiology solutions at national and international levels.

## Author Contributions

All authors contributed sufficiently and meaningfully to the conception, design, drafting, editing, and revising the manuscript.

## Author Disclaimer

DN-O, NA-M, and CH are staff members of the WHO. The authors alone are responsible for the views expressed in this article and they do not necessarily represent the decisions, policy, or views of the WHO.

## Conflict of Interest

The authors declare that the research was conducted in the absence of any commercial or financial relationships that could be construed as a potential conflict of interest.

## Publisher's Note

All claims expressed in this article are solely those of the authors and do not necessarily represent those of their affiliated organizations, or those of the publisher, the editors and the reviewers. Any product that may be evaluated in this article, or claim that may be made by its manufacturer, is not guaranteed or endorsed by the publisher.

## Footnotes

^1^ https://dl.acm.org/doi/10.1109/WI-IAT.2011.311

^2^ https://www.mdpi.com/2071-1050/12/7/2931/htm

^4^ https://www.nature.com/articles/s41562-017-0281-4

^5^ https://springerplus.springeropen.com/articles/10.1186/s40064-015-1279-x

^6^ https://www.nature.com/articles/s41599-018-0189-2

^7^ https://www.frontiersin.org/articles/10.3389/fpsyt.2020.589618/full

^8^ https://dl.acm.org/doi/10.1145/2597892
